# Succession of Fungal Communities at Different Developmental Stages of Cabernet Sauvignon Grapes From an Organic Vineyard in Xinjiang

**DOI:** 10.3389/fmicb.2021.718261

**Published:** 2021-08-31

**Authors:** Lihua Zhu, Tian Li, Xiaoyu Xu, Xuewei Shi, Bin Wang

**Affiliations:** Food College, Shihezi University, Shihezi, China

**Keywords:** Cabernet Sauvignon grape, fungi community, Illumina high-throughput sequencing, ripening process, organic vineyard

## Abstract

Fungi present on grape surface considerably impact grape growth and quality. However, information of the fungal community structures and dynamics on the worldwide cash crop, the Cabernet Sauvignon grape, from the budding to ripening stages remains limited. Here, we investigated the succession of fungal communities on Cabernet Sauvignon grapes from an organic vineyard in Xinjiang, China at different developmental stages *via* high-throughput sequencing combined with multivariate data analysis. In total, 439 fungal amplicon sequence variants (ASVs) from six phyla were identified. The fungal communities differed over the budding to the berry stages. Moreover, *Aspergillus*, *Malassezia*, *Metschnikowia*, and *Udeniomyces* were predominant during the unripe stage, whereas *Erysiphe*, *Cryptococcus*, *Vishniacozyma*, and *Cladosporium* were dominant in the ripe stages. Notably, *Vishniacozyma* was the most abundant genus, conserved in all development stages. Moreover, network analysis resulted in 171 edges—96 negative and 75 positive. Moreover, fungal genera such as *Vishniacozyma*, *Sporobolomyces*, *Aspergillus*, *Alternaria*, *Erysiphe*, *Toxicodendron*, and *Metschnikowia* were present in the hubs serving as the main connecting nodes. Extensive mutualistic interactions potentially occur among the fungi on the grape surface. In conclusion, the current study expounded the characteristics of the Cabernet Sauvignon grape fungal community during the plant growth process, and the results provided essential insights into the potential impacts of fungal communities on grape growth and health.

## Introduction

Wine is a traditional alcoholic beverage fermented from various fruits and vegetables, particularly grapes. According to the time of fermentation, grape varieties, and grape color, wine can be classified into red, rose, and white (Swami et al., 2014). Moreover, the classic dry red wine is the most popular wine worldwide, with the largest production and sales volumes. The grapes used to make dry red wine mainly include Cabernet Sauvignon (*Vitis vinifera* L.), Merlot, Cabernet Franc, and Syrah (Rajha et al., 2017). In particular, Cabernet Sauvignon, originating from the Bordeaux region of France (Bowers and Meredith, 1997), is currently the most famous and important red grape variety; it also is widely cultivated in China because of its strong adaptability and the premium-quality wines that it produces (Jiang and Zhang, 2012).

Yeast colonizing on grapes have been widely investigated for their impact on wine quality and flavor complexity (Capozzi et al., 2015). Research has also focused on a series of plant pathogenic fungi that affect grapes, including *Erysiphe necator*, *Botrytis cinerea*, and *Plamospara viticola*—the causative agents of grapevine powdery mildew, gray rot, and downy mildew, respectively. In addition, grapes may bear saprophytic molds such as *Aspergillus* spp., *Cladosporium* spp., and *Penicillium* spp., which are directly responsible for several grape rots and are indirectly involved in food spoilage because they produce mycotoxins (Martins et al., 2014). These fungi are unable to grow in wines, and their effect on wine quality is due to grape damage.

The grape berry surface is, nevertheless, an unstable habitat of microorganisms. Diversity and stability of the aforementioned indigenous yeasts on the surface of grape are strongly associated with numerous factors, such as vineyard geography (altitude, latitude, and longitude) (Gao et al., 2019), climatic conditions (rain, temperature, humidity, and maturity period) (Liu et al., 2019), grape variety (Zhang et al., 2019), and viticultural practice (herbicides, fertilizers, pesticides, and fungicides used) (Cadez et al., 2010). It is reported that the berry development process contributes to changes in the composition and structures of fungal populations on grapes (Carmichael et al., 2017; Kioroglou et al., 2019). In particular, microbial community, especially fermentative yeasts, significantly increased at harvest and not in the early fruit developmental stage (Renouf et al., 2005).

Xinjiang Uygur Autonomous Region of China is a world-renowned wine-producing area due to the unique climate and geographical characteristics. As the primary red grape cultivars, Cabernet Sauvignon has been widely used to ferment premium-quality red wines globally (Bowers and Meredith, 1997; Radovanović and Radovanović, 2010). Recent research has shown that indigenous microorganisms, especially fungal communities, are key in grapevine health and growth (Novello et al., 2017). Although microbial communities in the microenvironment and fermentation process have been investigated, fungal community succession during Cabernet Sauvignon grape development warrants further research.

In the present study, we investigated the dynamic changes in fungal communities during the ripening stages through high-throughput sequencing combined with multivariate data analysis. In particular, the fungal communities of Cabernet Sauvignon grapes from an organic vineyard in Xinjiang at different developmental stages were characterized. Our results lay the foundation for research on the fungal community on Cabernet Sauvignon grapes and provides insights into fungal community structures and their potential role in grape growth and health during its agricultural production.

## Materials and Methods

### Grape Sampling

Cabernet Sauvignon grapes used in this study were collected from the vineyard of the professional winery Great Wall Wine in Xinjiang, China (86°03′E, 45°19′N) in 2020. Because of a dry continental climate, grapes grown in this region have few diseases originating from microorganisms or pest. The study vineyard is commercially managed under the principles of organic farming by the winery. No chemical fertilizers or insecticides are applied for pest or disease control within the grape growing season, and weeds are controlled via manually weeding every month.

To evaluate changes in the fungal communities during the grape maturation process, Cabernet Sauvignon grape berries were collected using sterile scissors at the following development stages based on the modified Eichhorn–Lorenz (E-L) system (Coombe, 1995): before flowering (A, E-L stage 19), fruit set (B, E-L stage 27), berries pea size (C, E-L stage 31), berries still hard and green (D, E-L stage 33), berries begin to color (E, E-L stage 35), berries not quite ripe (F, E-L stage 37), and completely ripe (G, E-L stage 38). Three biological replicates were sampled for each developmental berry stage, and each replicate was collected from five sample points of the vineyard. Considering the heterogeneity of the tested grapes, the berries were collected from the upper, central, and lower part of cluster at each sample point, both from the sun-exposed and shaded side. In total, 21 wine grape samples were collected. These grape samples were placed in sterilized plastic bags and then into cool boxes, immediately shipped back to the laboratory, and stored at −80°C before molecular analysis.

### Genomic DNA Extraction and Amplification

After thawing for 30 min at 28∘C, 20 g of healthy, undamaged berries were placed in sterile flasks with 40 ml of sterile water and subjected to orbital shaking at 150 rpm for 1 h. The suspension was separated from the berries by vacuum filtration using a 0.22-μm filter. Next, the sediment filtered were used for the extraction of total genome DNA using the CTAB/SDS method (Yu et al., 2019). The DNA concentration and purity were monitored using 1% agarose gel electrophoresis and a NanoDrop 2000 Spectrophotometer. The DNA was diluted to l μg/μl with sterile water and used as a template for polymerase chain reaction (PCR) amplification. The internal transcribed spacer (ITS) primer pair—namely ITS1f (5′-CTTGGTCATTTAGAGGAAGTAA-3′) and ITS2 (5′-GCTGCGTTCTTCATCGATGC-3′)—was used to amplify the partial fungal ITS region to assess fungal communities (Hamad et al., 2017). All PCR reaction systems were conducted in using a 30 μl volume, including 15 μl of Phusion High-Fidelity PCR Master Mix (New England Biolabs), 0.2 μM of the forward and reverse primers, and approximately 10 ng of template DNA. Thermal cycler conditions consisted of an initial denaturation at 98∘C for 1 min, followed by 30 cycles of 98∘C for 10 s, 50∘C for 30 s, and 72∘C for 30 s and a final extension at 72∘C for 5 min.

### PCR Product Purification and Library Preparation

The PCR products were mixed with the same volume of 1× loading buffer (containing SYB green), which were then detected *via* 2% agarose gel electrophoresis. Then, the PCR products were mixed in equimolar ratios and purified using a Qiagen Gel Extraction Kit (Qiagen, Germany) (Zetsche et al., 2017). Sequencing libraries were generated using a TruSeq DNA PCR-Free Sample Preparation Kit (Illumina, United States) following the manufacturer’s recommendations, and index codes were added (Nones et al., 2014). The library quality was evaluated on a Qubit@2.0 Fluorometer (Thermo Scientific) and an Agilent Bioanalyzer 2100 system (Kao et al., 2016). Finally, the library preparations were sequenced on an Illumina NovaSeq platform with 250-bp paired-end reads generated.

### Bioinformatic Analyses

Paired-end reads were assigned to samples according to their unique barcodes and then truncated by removing barcodes and primer sequences. The paired-end reads were then merged using FLASH (Magoč and Salzberg, 2011). Quality filtering of the raw tags was performed under specific conditions to obtain the high-quality clean tags according to the QIIME quality control process (Bokulich et al., 2013). The tags were compared with a reference database using the UCHIME algorithm (Edgar et al., 2011) to detect chimera sequences, which were subsequently removed, and effective tags were obtained (Haas et al., 2011).

Sequence analyses of the clean tags were performed using Divisive Amplicon Denoising Algorithm 2 (DADA2; version 1.6.0) in R (version 3.3.4) (Callahan et al., 2016). Within DADA2, sequences were subjected to filtering, de-replication, and further filtering by using the sample inference algorithm and learned error rates within the DADA2 pipeline. The paired-end reads were merged using standard arguments, and chimeric sequences were filtered out.

Taxonomy was assigned using the Unite database (version 10.10.2017). The DADA2 method created an amplicon sequence variant [Amplicon sequence variants (ASVs); similar to operational taxonomic units, but with the ability to resolve amplicons to a single nucleotide], enabling species-level identification when 100% of the sequences matched the reference.

### Statistical Analyses

Operational taxonomic unit-level alpha diversity indices, including Chao1 richness estimator, Shannon diversity index, Simpson index, and Abundance-based Coverage Estimator (ACE) metric, were measured using the ASV table in QIIME. Rarefaction curves were plotted to investigate sequencing depth. The microbial community distribution patterns among different growth stages were analyzed using principal components analysis (PCA) based on beta diversity by using Simca (version 14.1). Amplicon sequence variants aggregates and intersections between the seven grape stages were visualized graphically on R (version 2.15.3) with the UpSet package. Hierarchical cluster analysis and heatmap construction were also performed using R (version 2.15.3) with the heatmap package. The similarities and differences in the composition and structures of the different samples are shown using the corrplot package in R. A linear discriminant analysis effect size (LEfSe) was applied to estimate discriminant fungal clades using The Huttenhower Lab website^1^. In addition, based on an LDA score (log10) of >2.0, the significantly enriched fungal groups were screened via linear discriminant analysis (LDA). Furthermore, network analysis based on Pearson’s correlation coefficient (*r*) of >0.8 or <−0.3 between two genera was performed and visualized using Gephi (version 0.9.2).

## Results

### Richness and Diversity Assessment

**FIGURE 1:**
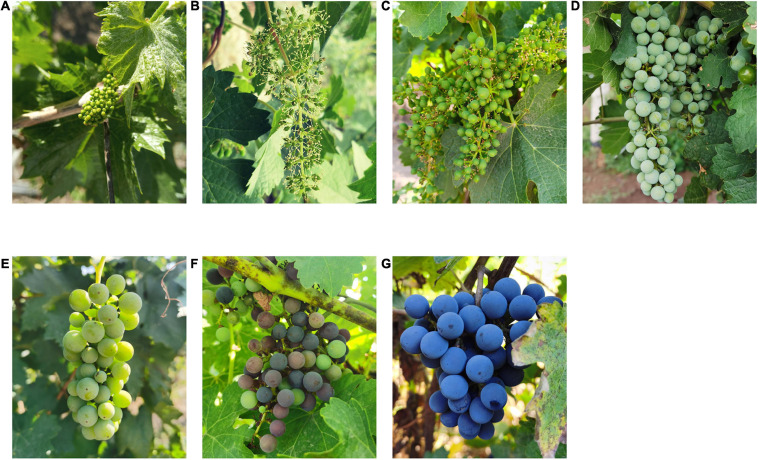
Appearance changes of Cabernet Sauvignon grape at different growth stages. **(A)** before flowering; **(B)** fruit set; **(C)** berries pea size; **(D)** berries still hard and green; **(E)** the beginning of the berry ripening; **(F)** berries not quite ripe; **(G)** harvest ripe.

High-throughput sequencing was used to assess fungal communities present on grape surface at different ripening stages (Figure 1). In total, 644,112 fungal sequences were obtained from 21 samples at seven different mature stages. Of the fungal sequences, 439 unique fungal ASVs were detected. The ASVs belonged to 6 phyla, 25 classes, 51 orders, 83 families, and 121 genera. The rarefaction curves tended to be flat, indicating that the sequencing results adopted were sufficient to reflect the whole fungal diversity (Supplementary Figure 1).

#### Alpha Diversity Analysis

The alpha diversity was analyzed using the Shannon, Simpson, ACE, Chao, and richness indices, which represent the fungal community richness and diversity (Table 1). Among the present diversity indices, the richness, Chao1, and ACE indices increased and then decreased after the growth process changed. Among all growth stages, the A stage had the highest Shannon index but the lowest Simpson diversity index, indicating a high diversity in a fungal community. In addition, compared with the samples from other developmental stages, D time points had higher-than-expected species community richness, based on the richness indices (e.g., richness, Chao1, and ACE), but not diversity, based on the Simpson index. The C stage had significantly greater richness than did the A and B stages, based on the richness, Chao, and ACE indices. However, the richness, Chao, and ACE index values for the A, B, F, and G stages were similar. Therefore, fungal community richness and diversity exhibited a correlation with the growth process, and differences were observed according to the alpha diversity indexes.

**TABLE 1:** Abundance and diversity indexes of fungi communities on the surface of wine grapes at seven different growth stages.

**Sample**	**Richness**	**Chao1**	**ACE**	**Shannon**	**Simpson**
A	95.33 ± 15.50	110.69 ± 23.98	115.01 ± 27.86	2.73 ± 0.08	0.14 ± 0.01
B	117.67 ± 21.94	183.51 ± 17.75	192.24 ± 14.19	2.40 ± 0.32	0.21 ± 0.08
C	162.67 ± 17.95	199.46 ± 20.23	207.40 ± 14.55	2.64 ± 0.14	0.18 ± 0.02
D	185.33 ± 2.29	229.74 ± 32.47	215.07 ± 20.64	2.46 ± 0.10	0.18 ± 0.02
E	154.00 ± 40.04	196.15 ± 43.86	201.91 ± 53.52	2.24 ± 0.20	0.22 ± 0.03
F	110.67 ± 6.03	153.25 ± 19.10	150.12 ± 8.25	2.11 ± 1.17	0.19 ± 0.01
G	129.00 ± 5.20	161.00 ± 10.99	171.14 ± 1.75	2.32 ± 0.06	0.14 ± 0.01

*The values in the table are shown as the mean ± standard deviation; the different lowercase letters express significant differences among different ripening stages in the 95% confidence interval. (A: before flowering; B: fruit set; C: berries pea size; D: berries still hard and green; E: the beginning of the berry ripening; F: berries not quite ripe; G: harvest ripe).*

#### Beta Diversity Analysis

**FIGURE 2:**
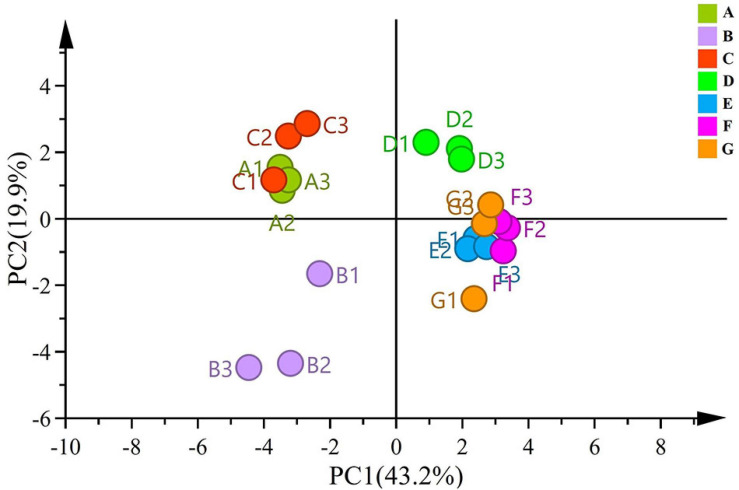
PCA plots based on unweighted UniFrac distance metrics for fungal communities.

To compare significant differences and understand clustering of samples between the groups, beta diversity was investigated *via* PCA. PC1 and PC2 accounted for 43.2% and 19.9% of the variance, respectively (Figure 2). The samples from the same developmental stages tended to cluster well together. In the PCA score plot, samples from different ripening stages could be divided into three different categories. Moreover, the score plot deriving from the PCA highlighted that A, B, and C stages were located at the negative side of PC1, whereas the B stage was located in the negative side of the PC2. All the sample groups were clustered together during the A and C stages, and the distance from the B stage was scattered—indicating that the differences were obvious between the B stage and the A and C stages. The four late time points (i.e., D, E, F, and G) related samples were clustered into one group located on the positive side of the PC1.

Notably, the D stage was found to be a key stage of diversity changes in grape-associated fungi. The PCA of grape-related fungal at different growth stages showed that different growth times may contribute to the differentiation of fungal composition. Moreover, the fungal community structure of the grape epidermis was gradually established at the D stage.

#### ASV-Based UpSet View

**FIGURE 3:**
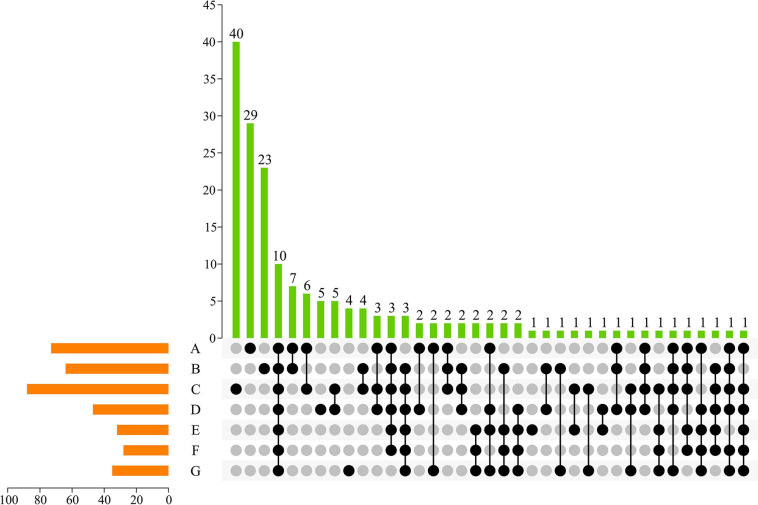
UpSet plot of ASVs of fungal communities in different growing periods. Dark circles indicated samples with containing accessions and connecting bar indicated multiple overlapping samples.

To visualize unique ASVs and those shared between different developmental stages, an UpSet plot was created (Figure 3). In an UpSet plot, a single point represents a unique species in each natural growth stage of grapes. According to the current results, 29, 23, 40, 5, 1, and 4 ASVs were uniquely present at the A, B, C, D, E, and G stages, respectively. Most ASVs (*n* = 40) on the skin of grapes were present at the C stage. At the same time, the C stage shared 6, 4, 5, and 1 fungal ASV with the A, B, D, and E stages, respectively. In total, 10 ASVs—accounting for 2.72% of the total ASVs—were shared by the samples from the seven growth periods, indicating that whole periods had a low similar level of fungal diversity. Two fungal ASVs were shared at the early developmental stages (i.e., A, B, and C stages), and two ASVs were shared at the later developmental stages (i.e., D, E, F, and G stages). Consequently, the differences of grape epiphytic fungal community structures were found to be primarily controlled by the grape mature stage.

### Dynamics of Fungal Community at Grapes Phenological Stages

**FIGURE 4:**
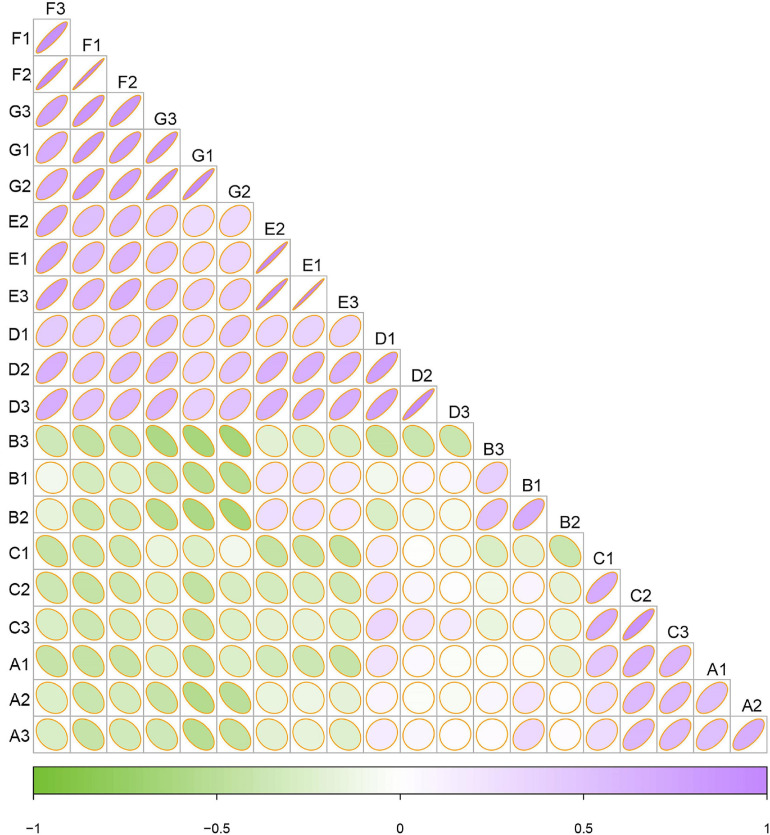
Heatmap of fungal communities based on Bray–Curtis distance indices. Clustering of samples based on Bray–Curtis distance indices was calculated by ASVs at a distance of 3%. Color from green to purple indicated increased similarity. The radian of the fan corresponds to the correlation.

To examine the similarities and differences between samples from different developmental stages, a correlation heatmap was generated on the basis of the Bray–Curtis distance (Figure 4). Systematic cluster analysis illustrated that the correlation of samples collected between the three early growth stages (i.e., A, B, and C) and the four later growth stages (i.e., D, E, F, and G) was small, whereas the grape samples collected within the same or similar ripening stage had relatively strong correlations. The results showed that the correlation of the fungal community in the late developmental stage was higher than that in the early developmental stage.

**FIGURE 5:**
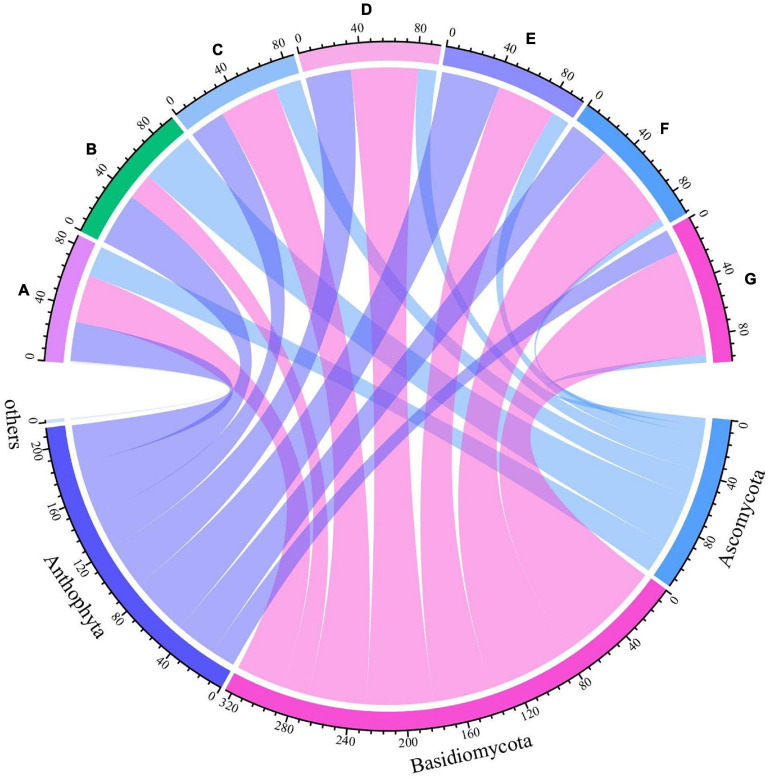
Differences in fungal communities on Cabernet Sauvignon grapes at different growth stages at the phylum levels.

**FIGURE 6:**
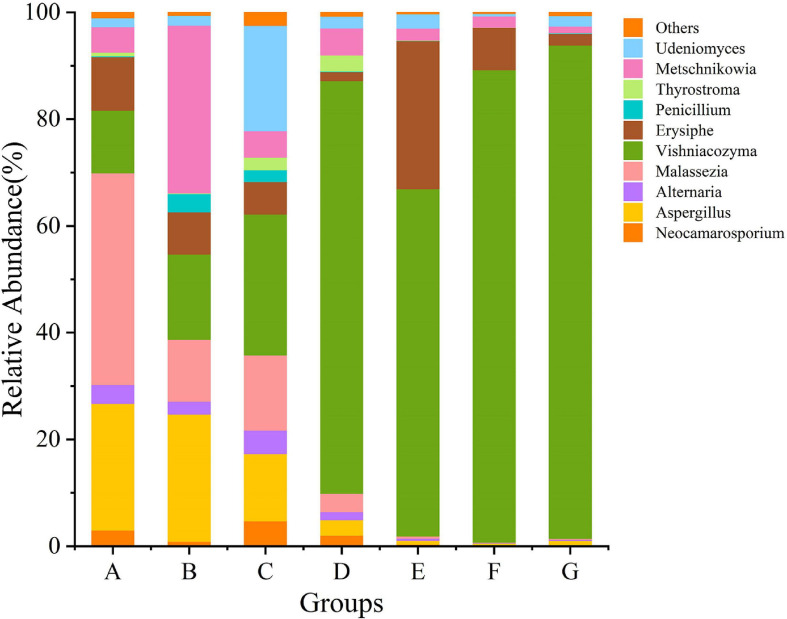
The relative abundance of fungal communities at the genus level.

Grape fungal community compositions evolved over time. At the phylum level, most significant was the massive colonization of the grapes by *Basidiomycota*, which increased as the development stage proceeded; their abundance reached 77% at the G stage. The second highest abundance was shown by *Anthophyta* (43%) in the E stage. In contrast, the low abundance was demonstrated by *Ascomycota* (41%) in the B stage (Figure 5 and Supplementary Figure 2). At the genus level, the dominant taxa found included representatives of the genera *Neocamarosporium*, *Aspergillus*, *Alternaria*, *Malassezia*, *Vishniacozyma*, *Erysiphe*, *Penicillium*, *Thyrostroma*, *Metschnikowia*, and *Udeniomyces* (Figure 6). Of these, *Penicillium* and *Thyrostroma* mainly appeared at the early phenological stages and finally became undetectable at harvest, whereas *Vishniacozyma* remained the most abundant yeast in grape berries along with several grape spoilage fungi such as *Erysiphe* and *Aspergillus* throughout all the developmental stages. The relative abundances of *Erysiphe* reached a maximum (28%) in the E stage and a minimum (1%) in the G stage. Notably, the relative abundance of *Vishniacozyma* tended to increase and reach a maximum at harvest time. In contrast, the abundance of *Malassezia and Aspergillus* decreased during grape ripening, and these fungi were more highly abundant at the first ripening stage that at other stages.

Some of the fungi detected here—namely, *Alternaria*, *Metschnikowia*, and *Udeniomyces*—have been previously described to be present on grape surfaces; some of them are even known to be inhabitants of grapes. The results showed that the yeast diversity generally tends to be poor, and the dominant species become increasingly prominent as the berries’ expansion and development progresses.

### Cluster Analysis

**FIGURE 7:**
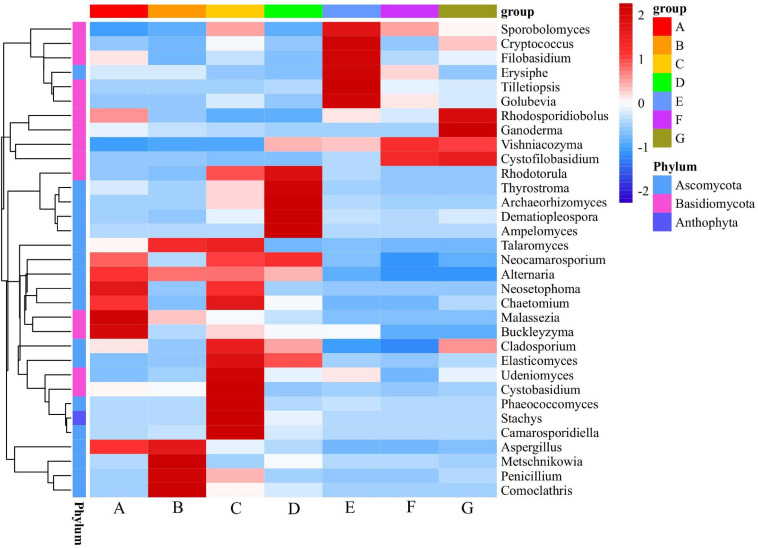
Heatmap of top 35 abundant genera of fungal communities at different developmental stages. Samples are clustered according to the similarity among their constituents and arranged in a horizontal order. In the figure, red represents the more abundant genera in the corresponding group and blue represents the less abundant genera.

Using the species annotation and abundance results obtained from all the samples at the genetic level, the genus with the top 35 abundance were selected; the species and samples were then clustered based on the abundance information of samples and a heatmap was generated (Figure 7). The use of a heatmap allows for intuitively determining changing patterns are more or less concentrated in which taxa (or group) in the samples. The composition of fungi in the samples at different sampling times differed considerably. A large gap between the C stage and the other stages was reflected by the color gradient and similarity. Moreover, most of the identified genera belonged to the phyla of *Ascomycota* and *Basidiomycota*. The D stage had the highest alpha diversity index (the richness, Chao1, and ACE index), containing mainly *Rhodotorula*, *Thyrostroma*, *Archaeorhizomyces*, *Dematiopleospora*, and *Ampelomyces*. *Malassezia*, *Aspergillus*, *Buckleyzyma*, *Chaetomium*, *Neoetophoma*, *Alternaria*, and *Neocamarosporium* were the dominant genera at the A stage. At the C stage, *Camarosporidiella*, *Stachys*, *Phaeococcomyces*, *Cystobasidium*, *Elasticomyces*, *Cladosporium*, *Chaetomium*, *Neocamarosporium*, *Alternaria*, and *Undeniomyces* were the primary genera. At the E stage, the most abundant sequences were related to *Ampelomyces*, *Dematiopleospora*, *Archaeorhizomyces*, *Throstroma*, and *Rhodotorula*. Notably, *Cystofilobasidium*, *Vishniacozyma*, *Rhodosporidiobolu*, and *Ganoderma* were dominant in the G stage.

### Biomarker Discovery

**FIGURE 8:**
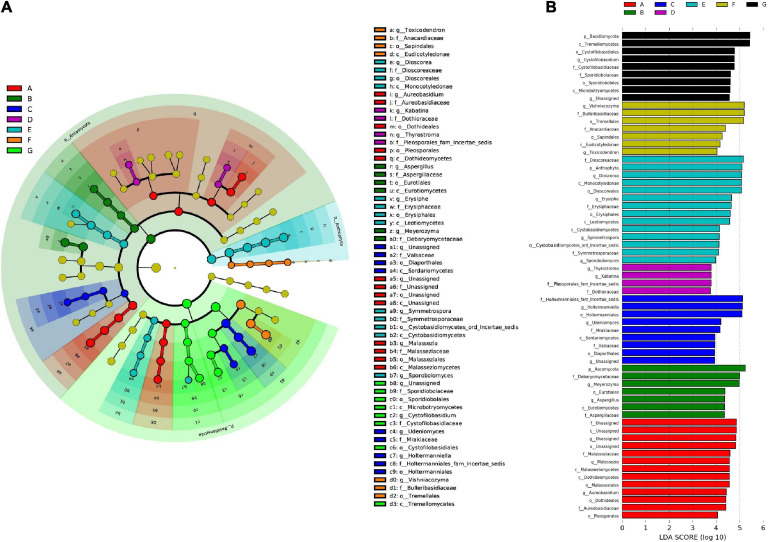
Linear discriminant analysis (LDA) and effect size (LEfSe). **(A)** The bar charts report the taxonomic representation statistically and biologically as determined by LEfSe analysis; **(B)** the linear discriminant analysis (LDA) value distribution histogram.

Here, we used the LDA effect size (LEfSe) to recognize the significant biomarkers among fungal taxa in different phenological stages of Cabernet Sauvignon grapes. The LEfSe of all species demonstrated 63 fungal taxa with significant differences among the different developmental stages at an LDA threshold of 2.0, namely, 3 phyla, 11 classes, 14 orders, 17 families, and 18 genera (Figure 8). In total, 13 fungal classes were significantly enriched in the A stage—including *Malasseziomycetes*, *Dothideomycetes*, and *Aureobasidium*. Seven fungal classes were significantly enriched at the B stage—including *Ascomycota*, *Aspergillaceae*, and *Eurotiales*. Nine fungal groups were significantly enriched at the C stage—including *Valsaceae* and *Holtermanniales*. Four fungal groups were significantly enriched at the D stage. Moreover, 14 fungal groups were significantly enriched at the E stage—including *Anthophyta*, *Dioscoreales*, and *Cystobasidiomycetes*—and 7 fungal groups were significantly enriched at the F stage. Nine fungal groups were significantly enriched at the G stage—including *Basidiomycota*, *Cystofilobasidiales*, and *Tremellomycetes*.

Thus, from the flowering begin to mature stages, wine grape microbiota can be distinguished at least at the phylum and class levels by their demonstrative microorganisms—with the exception of the D stage, where the distinction is limited to the family level.

### Co-occurrence Analyses for Relationships Among Different Microbes

**FIGURE 9:**
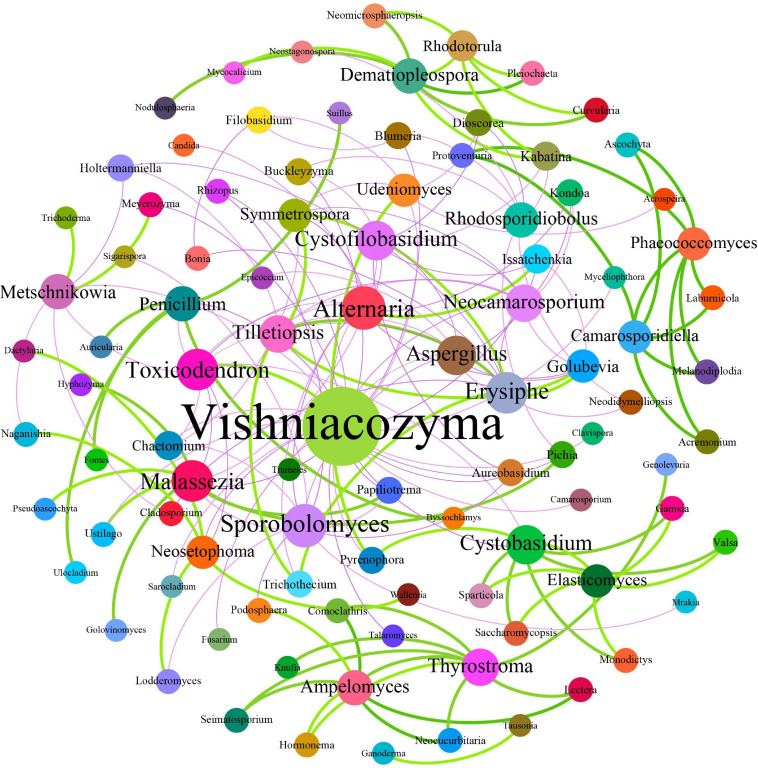
Co-occurrence network among fungi at the genus level. Each dot in the figure represents a species, and the size of the dot indicates the number of related relationships with other species. Solid lines indicate positive correlations; dash lines indicate negative correlations.

Fungal interactions—usually reflected by co-occurrence correlations—are considered an important factor underpinning the fungal community structures. In the present study, Pearson’s correlation coefficients were estimated to investigate the potential beneficial or antagonistic relationships among different fungal genera of grape surfaces. In total, 171 dominant fungal genera were used for co-occurrence network analysis after the unclassified and relatively low abundance genera were removed (Figure 9). The generated networks consisted of 96 nodes and 171 edges; here, the nodes represented fungal genera, and the edges represented the positive (green, Pearson’s *r* > 0.8) or negative (purple, Pearson’s *r* <−0.3) correlations between pairs of genera. The larger the size of the node, the more important is the genera in the fungal community. Based on the network connectivity statistics, fungal genera such as *Vishniacozyma*, *Sporobolomyces*, *Aspergillus*, *Alternaria*, *Erysiphe*, *Toxicodendron*, and *Metschnikowia* were among the hubs that served as the main connecting nodes. Simultaneously, *Vishniacozyma*, *Udeniomyces*, *Aspergillus*, and *Alternaria* were negatively correlated with almost all other fungal genera, whereas *Ampelomyces*, *Rhodotorula*, and *Dematiopleospora* were positively correlated with almost all other fungal genera. In addition, *Metschnikowia* was negatively correlated with *Naganishia* but positively correlated with *Trichoderma*. Notably, the correlation analysis results indicated that *Erysiphe* was correlated positively with *Golubevia*, *Symmetrospora*, and *Tillctiopsis* but negatively with *Neocamarosporium* and *Aspergillus.* A positive correlation was observed between *Pichia* and *Malassezia*.

## Discussion

A considerable amount of attention has been focused on the microbial communities associated with wine grapes. However, the changes in the microorganisms inhabiting the grape surface are dynamic over space and time. Previous research has only focused on the harvest-to-ripe stages with a culture-dependent approach; consequently, little is known about the diversity and dynamics among epiphytic fungal communities present on wine grape surfaces across their developmental stages. The culture-independent microbiota studies have provided a reference framework for microbial population diversity and composition on wine grapes and captured the temporal fungal microbial shifts during the phenological stages—as a result increasing the understanding of grape-associated microbial communities. The current study is a very important step into further evaluation of the contributions of microorganisms in the functioning of the vineyard ecosystem and the expansion of potential uses of specific microorganisms to influence grape growth and health.

In general, alpha and beta diversity metrics of wine grape microorganisms have demonstrated that the epiphytic fungal communities are significantly impacted by the mature stage. One hypothesis is that the changes in microbial community are related to the changes in the surface compositions of the grape berries, which include differences in tissue pH and carbon and nitrogen compound contents as well as external factors such as pollen deposits, various organic honeydews, and debris (Janisiewicz et al., 2010; Martins et al., 2014). However, one should not rule out that fungal communities are not randomly combined, but communities with different habitat patterns on the basis of the developmental stages of grapevines.

In the present study, throughout the whole developmental period, the fungal diversity remained relatively constant, even though the number of species tended to decrease. In contrast, Liu and Howell (2021) observed that after the fruit set stage, the fungi diversity of grape berries significantly increased over the remaining ripening process. However, the reasons for these changes warrant investigation. In general, as demonstrated for grape epiphytes (Jara et al., 2016), the variety of viticulture areas may influence the microbial terroir. Therefore, due to environmental and region fluctuations, only 10 core ASVs of fungal communities continue to exist over time.

Studies have shown that the structural and functional diversity of microbial communities on a wide range of plants (such as *Arabidopsis*, *Medicago*, wheat, maize, pea, and sugar beet) change according to the plant ripening stages (Mougel et al., 2006; Houlden et al., 2008; Micallef et al., 2009). Similarly, in our study, the succession of the microbiome was related to the grape development stages and a “vineyard-specific” distribution was noticed at each stage. This may be the response of grape-related microorganisms to plant development and changing environment. Most distinct community structures of datasets were observed at berry size (Figure 3). At the berries still hard and green stage, the grapevine showed highly distinct changes in fungal community diversity (Figure 2). Nevertheless, many factors are likely to explain the observed differences.

In grapevine phenology, wine grapes undergo various major biochemical and physiological changes (Conde et al., 2007), which may affect the structure of fungal communities inhabiting a heterogeneous microenvironment and determine the grape quality at harvest. The flowering begin stage is mainly characterized by high ecological interaction with other organisms (such as pollinating insects) (Becher et al., 2018; Zhang et al., 2020). Yeasts such as *Metschnikowia* colonize flowers and produce distinctive scent profiles to enhance floral signaling (Lachance et al., 2001; Becher et al., 2018). The berry stages, such as the C stage, are mainly characterized by physical and chemical changes, such as rapid berry growth both through cell division and expansion (Sun and Xiao, 2015) and increased levels of phenolic compounds because of tartaric acid accumulation (Cuadros-Inostroza et al., 2016). All these factors may have created a more favorable environment for microbial colonization in grapes, and microbial communities became richer at this stage compared with other developmental stages. In particular, *Rhodotorula* that surfaced at this time point demonstrated plant growth-promoting capabilities (Saha and Seal, 2015).

The veraison stage is accompanied by changes on berry color and reduced growth. When the grape is ripe, it shows the greatest size, elasticity, and sugar accumulation (Ben-Hong et al., 2011). In this stage, yeasts likely have the largest surface area available for adherence and probably increased access to nutrients. In our study, the increased abundance of *Cryptococcus*, *Vishniacozyma*, and *Cladosporium* in the G stage suggests that these fungi can withstand high sugar content and low moisture. A study reported that plants can recruit microbes to participate in the key physiological processes and drive microbial assemblages to respond to biotic or abiotic stresses and improve environmental fitness (Turner et al., 2013). Our data may indicate how plant-driven microbes respond to environmental changes.

We also identified characteristic dominant genera typically linked with wine and grape (i.e., *Vishniacozyma, Metschnikowia*, and *Malassezia*) along with several pathogenic fungal genera (*Aspergillus*, *Penicillium*, and *Erysisphe*) (Lorenzini et al., 2016). *Vishniacozyma* was present in all mature stages but was negatively correlated with almost all other fungal genera; this may be because it shows relatively better adaptation to the changes in the grape environment as well as aids suppression and elimination of other characteristic microorganisms, resulting in strong selection (Barata et al., 2012). *Vishniacozyma* is a cosmopolitan yeast and has been isolated from several substrates, such as soil (including Antarctic, Alaska, and Arctic soils) (Tsuji et al., 2019), as well as from wood (Félix et al., 2020) and cold environments (surface of Vidal grapes) (Shi et al., 2020). Nevertheless, its possible impacts on wine quality remain unknown and warrant further investigation, especially with regard to its effects on wine flavor. Furthermore, *Vishniacozyma* have biocontrol effects on the blue molds and gray molds that infect pears (Lutz et al., 2013); our results also suggest a possible antagonistic effect between *Vishniacozyma* and *Erysisphe*. The biocontrol potential of *Vishniacozyma* for grape powdery mildew requires further analysis.

Notably, the abundance of *Aureobasidium* was low in our study, whereas it was the predominant genus in grapes in other studies (Grube et al., 2011; Zhang et al., 2019). In addition, *A. pullulans* have the capacity to adapt well to saprophytes on grape berries; however, it is not widely distributed in Shangri-La and other parts of China (Li et al., 2010; Zhao et al., 2021). In the current study, we detected *Erysisphe*, the richness of which was relatively high in the E stage, which then declined sharply—from 28% in July to 1% in September. This finding is consistent with an earlier report that grape powdery mildew, one of the most harmful fungal diseases, usually occurs in July (Han et al., 2016). This also indicates that grape powdery mildew is quite serious in this area and appropriate preventive measures need to be taken (Amrine et al., 2015). Notably, *B, cinerea*, a well-known necrotrophic fungal pathogen of grapes (Jaspers et al., 2013), was not detected in the current study, possibly due to geographical location (Rivera et al., 2013). This also illustrates that a healthy microbial composition should keep the pathogenic populations at a low or even undetectable levels; this aids in preventing harmful plant diseases.

The relative abundance of *Penicillium* was also high in the B stage (Figure 7). Although some *Penicillium* species can cause severe damage to crop, many of them have been reported to be beneficial—preventing plant diseases and inhibiting pathogen activity (Van Wees et al., 2008). *Aspergillus*, which enriches at the early development stages, is a predominant global wine contaminant; it is reported to produce ochratoxin A, which causes human health hazards (Ferrari et al., 2017). Notably, in some organisms, *Alternaria*, considered one of the main mycobiota populations of grapes at harvest, gradually reduces during the berry ripening process (Tournas and Katsoudas, 2005). In particular, *Alternaria* can infect fruit during flowering and immature development by behaving as a biological nutritional pathogen and remaining latent in the outermost layer of the fruit, waiting for suitable conditions to become conducive to disease expression (Prusky et al., 2013). However, contrasting findings indicate that species of *Alternaria* can control the growth of different pathogens such as *Rhizoctonia solani*, *Fusarium oxysporum*, *B. cinerea*, and *Pseudomonas aeruginosa* (Yu et al., 2011; Ortega et al., 2020).

Biocontrol microbes may provide a sustainable alternative to chemical control of pathogens (Lecomte et al., 2016). In the current study, the relative abundance of *Metschnikowia* and *Rhodotorula* was relatively high in the D stage and that of *Ampelomyces* was relatively high in the B stage (Figure 7). These three fungi have beneficial antifungal properties; *Metschnikowia*, *Rhodotorula*, and *Ampelomyces* control common fungal pathogens that cause apple ring rot, cucumber powdery mildew, and grape gray mold blight, respectively (Tian et al., 2004; Zhang et al., 2019). *Cryptococcus*—an effective potential biocontrol agent effective against postharvest pathogens present on fruits (Renouf et al., 2005; Liu et al., 2013; Taylor et al., 2014)—was found predominantly in the E stage.

Taken together, these findings suggest that the beneficial microorganisms and plant pathogens can thrive in a balanced microbial ecosystem. Thus, identifying potential beneficial strains and antagonistic strains that control pathogenic infections is essential.

In conclusion, our study provides in-depth information regarding the differences in the fungal communities on the surface of grapes during the developmental stages. The results illustrate how the fungal microbiota increase in size and how their structure changes during the ripening process. Furthermore, the unclassified microbiota detected in the current study confirmed that the classification of the grape-related fungi needs further exploration and documentation.

In addition, future studies should thus focus on preharvest management practices that can increase the natural abundance of potential biocontrol communities, such as those of *Cryptococcus*, *Vishniacozyma*, and yeast-like fungus. This may result in reduced pesticide costs and postharvest losses due to rot.

In summary, increasing our understanding of microbial ecology and community dynamic change in the vineyard throughout the developmental stages can help improve management techniques for maintaining and producing healthy, high-quality grapes and allowing the expression of the regional character of the wine.

## Data Availability Statement

The data presented in the study are deposited in the NCBI repository, accession number PRJNA750856.

## Author Contributions

LZ collected wine grape samples, compiled the figures and table, and wrote the manuscript. TL and XX conceived the framework of the manuscript. BW conducted the bioinformatic analysis of data and provided advice and constructive critiques. XS supervised the research activities. All authors reviewed the manuscript.

## Conflict of Interest

The authors declare that the research was conducted in the absence of any commercial or financial relationships that could be construed as a potential conflict of interest.

## Publisher’s Note

All claims expressed in this article are solely those of the authors and do not necessarily represent those of their affiliated organizations, or those of the publisher, the editors and the reviewers. Any product that may be evaluated in this article, or claim that may be made by its manufacturer, is not guaranteed or endorsed by the publisher.

## Footnotes

1
http://huttenhower.org/galaxy
