# Supplementary material for: Succession of Fungal Communities at Different Developmental Stages of Cabernet Sauvignon Grapes From an Organic Vineyard in Xinjiang
Source: Front Microbiol. 2021 Aug 31;12:718261. doi: 10.3389/fmicb.2021.718261 (PMC8439140; doi:10.3389/fmicb.2021.718261)
Supplement: Supplementary file 1 [file Data_Sheet_1.PDF]

Supplementary Information for

Succession of Fungal Communities at Different Developmental Stages of Cabernet Sauvignon Grapes from an Organic Vineyard in Xinjiang

Lihua Zhu, Tian Li, Xiaoyu Xu, Xuewei Shi*, Bin Wang*

1. Food college, Shihezi University, Shihezi 832000, Xinjiang Uygur Autonomous Region, P. R. China.

* Corresponding authors

E-mail addresses: B. W. : binwang0228@shzu.edu.cn; X. S. : shixuewei@shzu.edu.cn

Tel.: 86-0993-2058093

Contents

Supplementary Figures 3
Fig. S1. Rarefaction curves of ASVs (amplicon sequence variants) clustered at 97% phylotype similarity level of wine grape samples at seven different growth stages for fungal communities. 3
Fig. S2. The relative abundance of fungal communities at the phylum level 4

Supplementary Figures

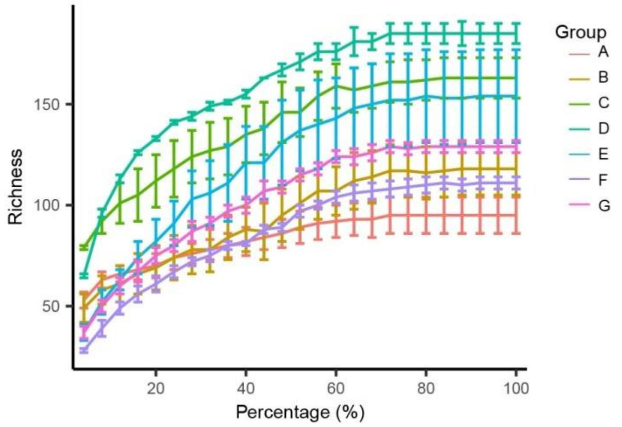

Fig. S1. Rarefaction curves of ASVs (amplicon sequence variants) clustered at 97% phylotype similarity level of wine grape samples at seven different growth stages for fungal communities.

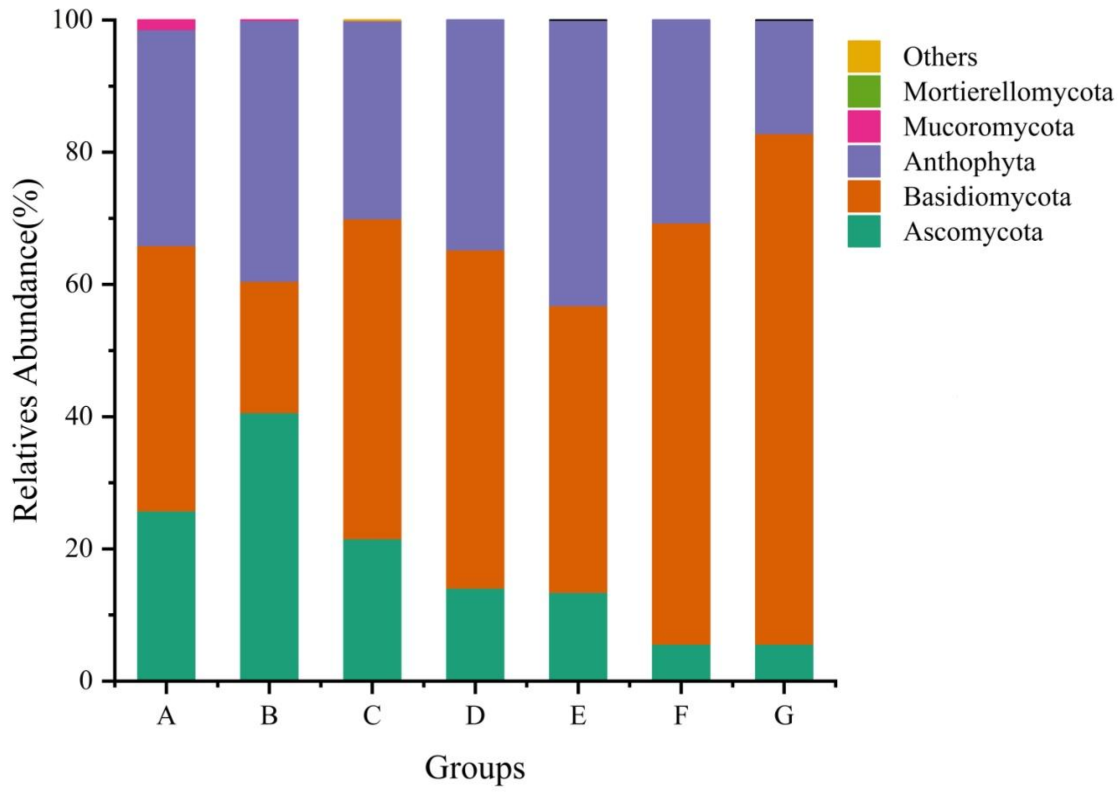

Fig. S2. The relative abundance of fungal communities at the phylum level
